# Lactiplantibacillus Plantarum GUANKE Alleviates Allergic Asthma by Restricting Monocyte‐to‐DC Differentiation Via Microbiota‐Associated IAA

**DOI:** 10.1002/advs.202523466

**Published:** 2026-09-16

**Authors:** Yujia He, Jielan Mi, Zhiping Zhao, Xiao Zhang, Zhihan Yang, Kun Yue, Yuanming Huang, Liqiong Song, Xiaoting Wen, Jianguo Xu, Zhihong Ren

**Affiliations:** ^1^ National Key Laboratory of Intelligent Tracking and Forecasting for Infectious Diseases National Institute for Communicable Disease Control and Prevention Chinese Center for Disease Control and Prevention & Chinese Academy of Preventive Medicine Beijing P. R. China; ^2^ Institute of Public Health School of Medicine Nankai University Tianjin P. R. China; ^3^ Shanxi Bethune Hospital Shanxi Academy of Medical Sciences Third Hospital of Shanxi Medical University Tongji Shanxi Hospital Taiyuan P. R. China; ^4^ Gansu University of Chinese Medicine Gansu P. R. China; ^5^ Research Unit for Unknown Microbe Institute of Pathogen Biology Chinese Academy of Medical Sciences Chinese Academy of Medical Sciences & Peking Union Medical College Beijing P. R. China

**Keywords:** asthma, CX3CR1, dendritic cell, indole‐3‐acetic acid, NOTCH4

## Abstract

Emerging evidence highlights the role of the gut microbiota in allergic asthma, with microbial metabolites mediating distal immune responses. This study investigated the protective efficacy of *Lactiplantibacillus plantarum* GUANKE and the mechanisms associated with dendritic cell (DC)‐driven allergic airway inflammation (AAI). GUANKE conferred both prophylactic and therapeutic protection by attenuating airway hyperresponsiveness and Th2‐driven inflammation while partially restoring regulatory T cell (Treg) population. Antibiotic depletion and fecal microbiota transfer indicated that GUANKE‐mediated protection was associated with remodeling of the indigenous microbiota and microbiota‐dependent restoration of circulating indole‐3‐acetic acid (IAA). Targeted serum metabolomics further revealed lower circulating IAA levels in patients with asthma than in healthy controls. Exogenous IAA reduced CX3CR1^+^Ly6C^−^ monocytic precursors and pulmonary CD11b^+^ DC accumulation. These reductions were preserved after CD11c‐specific aryl hydrocarbon receptor (AhR) deletion and accompanied by reduced JAK3‐STAT3 signaling, lower STAT3 occupancy at the *Jag1* promoter, and attenuation of the Jag1‐Notch4‐Hey1 axis. Pharmacological STAT3 activation or Jagged1‐mediated Notch reactivation partially reversed the effects of IAA. Collectively, these findings link microbiota‐associated IAA restoration to GUANKE‐mediated protection and implicate STAT3‐Jag1‐Notch4 signaling in monocyte‐to‐DC differentiation during AAI.

## Graphical abstract

*Lactiplantibacillus plantarum* GUANKE remodels the indigenous gut microbiota and is associated with microbiota‐dependent restoration of circulating IAA. IAA treatment attenuates JAK3‐STAT3 and Jag1‐Notch4‐Hey1 signaling and reduces CX3CR1^+^Ly6C^−^ monocytic precursor generation and pulmonary CD11b^+^ DC accumulation, together with reduced Th2 inflammation and partial restoration of the Treg population.

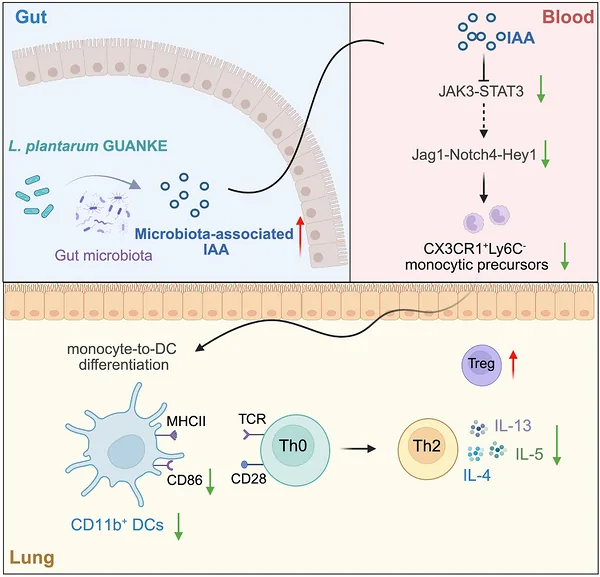

## 1 Introduction

Allergic asthma, the most common endotype of asthma, is characterized by reversible airflow obstruction, mucus overproduction, bronchial remodeling, and eosinophilic airway inflammation driven by T helper 2 (Th2) cell‐dominant inflammatory responses. The global prevalence of asthma continues to rise, with projections estimating 275 million affected individuals by 2050 [1]. As the major antigen‐presenting cells (APCs) in the lung, DCs capture and present inhaled allergens and initiate Th2 polarization. Th2‐derived cytokines, including IL‐4, IL‐5, and IL‐13, promote eosinophilic inflammation, mucus production, and B‐cell class switching, thereby facilitating allergen‐specific IgE production [2, 3].

The pivotal role of DCs in allergic asthma has been extensively studied in the past decades. Based on the distinct development and function, DCs have been classified into CD103^+^ and CD11b^+^ subsets [4, 5]. During allergic asthma, CD11b^+^ DCs are continuously recruited to inflammatory site to internalize antigen, mature and subsequently migrate to the draining mediastinal lymph nodes (MLNs) of the lung, where they promote Th2 cell polarization to exacerbate airway inflammation [6, 7]. Given the critical role of CD11b^+^ DCs in initiating and maintaining type 2 allergic airway inflammation (AAI), targeting their recruitment or activation may represent a promising therapeutic approach for allergic asthma.

Previous studies have demonstrated that CD11b^+^ DCs in the lungs of murine allergic asthma models are partially derived from circulating monocytes [8, 9]. These monocyte‐derived CD11b^+^ DCs share several surface markers with monocytes/macrophages, including CX3CR1, SIRP‐α, and CD11b. CX3CR1, the receptor for fractalkine, exhibits cell type‐specific expression patterns across various tissues. Among peripheral leukocytes, CX3CR1 is predominantly expressed on monocytes and plays a critical role in mediating the recruitment of circulating monocytes toward the pulmonary CD11b^+^ DC pool [10]. Specifically, Ly6C^low^CCR2^low^ monocytes rely on CX3CR1 signaling to maintain cellular survival, thereby replenishing the CD11b^high^ DC population in the lung [11]. Consistent with these findings, CX3CR1 has been demonstrated to facilitate the migration of pre‐DCs to the lung in the steady state [12]. Given the crucial role of DCs in orchestrating immune responses within the lung, these findings collectively highlight a close association between CX3CR1 expression and the migration, localization, and terminal differentiation of CD11b^+^ DC precursors within the lung. Importantly, genetic association studies have further implicated CX3CR1 in asthma pathogenesis, linking specific common SNPs within the CX3CR1 locus to an increased susceptibility to asthma [13], suggesting its potential significance in this prevalent airway inflammation.

Gut microbiota and its derivatives play a crucial role in modulating allergic asthma via the gut–lung axis [14]. Microbially derived indole metabolites (e.g., indole‐3‐acetic acid, indole‐3‐propionic acid, indole‐3‐lactic acid, and indole‐3‐butyric acid), produced by metabolism of tryptophan, exhibit altered levels in patients with allergic asthma [15, 16, 17]. As a key microbial metabolite, indole‐3‐acetic acid (IAA) has emerged as a compelling therapeutic target due to its remarkable anti‐inflammatory and immunomodulatory potential. Notably, hydrogen‐rich water‐induced elevation of IAA has been demonstrated to correlate strongly with its protective effects against allergic asthma [15]. Moreover, intraperitoneal administration of IAA significantly improves survival rates, body weight, and asthma symptom scores in OVA‐induced murine asthma models, while concurrently reducing inflammatory cell infiltration in lung tissue [18]. Nevertheless, the specific immune cell types targeted by IAA and the precise mechanisms underlying its actions in allergic asthma remain elusive and warrant further investigation.

Our previous study has shown that *Lactiplantibacillus plantarum* GUANKE, isolated from the feces of healthy individuals, enhances DC‐directed Th1 polarization and protects mice against influenza infection [19]. In the current study, we investigated whether GUANKE alleviates AAI through microbiota‐associated metabolic regulation in DC development. We focused on the contribution of the indigenous gut microbiota and IAA to GUANKE‐mediated protection, particularly the generation of CX3CR1^+^Ly6C^−^ monocytic precursors and replenishment of pulmonary CD11b^+^ DCs. Circulating IAA levels were also assessed in patients with asthma to validate the clinical relevance of IAA‐related metabolic alterations. Together, these analyses aimed to clarify how probiotic modulation of microbial metabolism influences DC‐driven allergic airway inflammation across the gut‐lung axis.

## 2 Methods

### 2.1 Human Subjects

In this study, patients with asthma (*n* = 28) and healthy controls (*n* = 28) were recruited from Shanxi Bethune Hospital (Shanxi, China) between October 2025 and June 2026. The clinical characteristics of the participants are provided in Table S1. Asthma was diagnosed according to the GINA guidelines. Written informed consent was obtained from all participants before sample collection, and the study was approved by the Medical Ethics Committee of Shanxi Bethune Hospital (LYLL‐2025‐004/PJ55).

### 2.2 Bacterial Strains and Preparation

The *L. plantarum* GUANKE strain, isolated from the feces of healthy individuals, has been deposited in the China General Microbiological Culture Collection Center (CGMCC) under the accession number CGMCC NO.21720. The genome sequence of GUANKE has been deposited in the NCBI database under the accession number SAMN05771125. GUANKE was cultured for 24 h in sterilized De Man Rogosa and Sharpe (MRS) medium at 37°C. Cultures were collected in the log phase, washed twice with sterile anaerobic PBS, and diluted to 10^9^ colony‐forming units (CFU) before gavage.

### 2.3 Animals

Female wild‐type (WT) BALB/c mice aged 4–6 weeks obtained from Beijing Vital River (Certificate No.: SCXK (Beijing) 2021‐0006, China) were housed under specific pathogen‐free (SPF) conditions. CD11c‐specific *aryl hydrocarbon receptor* knockout mice (*CD11c‐Cre* ^+^ *AhR* ^fl^/^fl^) were kindly provided by Dr. Shu Zhu from the Department of Veterinary Medicine, College of Animal Sciences, Zhejiang University (Hangzhou, China). Mice were maintained on a typical 12‐h light/dark cycle with free access to water. The experimental protocol was approved by the Institutional Animal Care and Use Committee in the Chinese Center for Disease Control and Prevention Laboratory Animal Center (Approval number: 2024–032). Mice were euthanized by CO_2_ inhalation, followed by cardiac puncture to ensure death.

### 2.4 OVA‐Induced Asthma Model (Establishment of Various Mouse Models)

**FIGURE 1:**
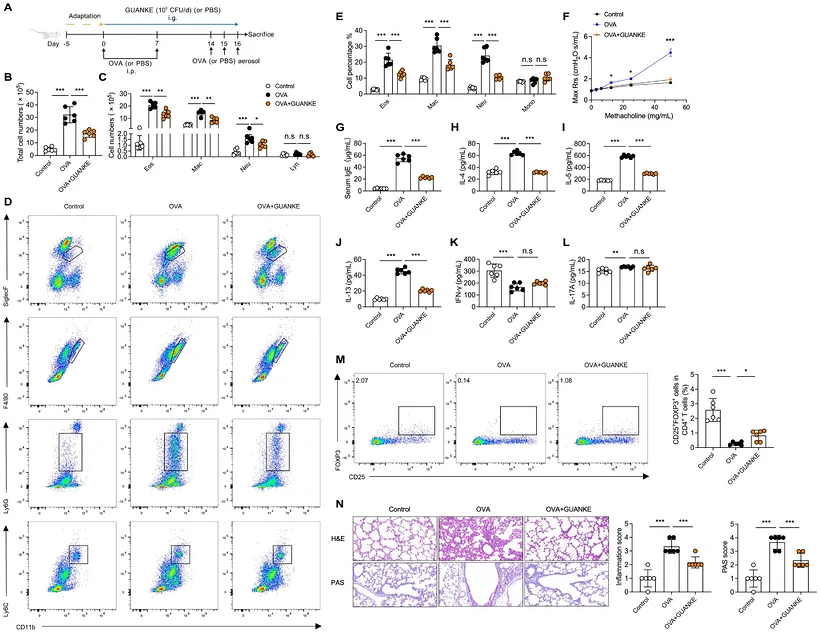
GUANKE administration alleviated airway inflammation in asthma. (A) Experimental scheme for B‐N. Mice were treated with GUANKE (1×10^9^ CFU) or PBS by daily gavage for 16 days, sensitized with OVA or PBS via intraperitoneal (i.p.) injection on days 0 and 7, and challenged with OVA or PBS aerosol from day 14 to day 16. (B) Total cell counts were determined in the BALF of mice. (C) Number of eosinophils (Eos), macrophages (Mac), neutrophils (Neu), and lymphocytes (Lyn) in BALF. (D) Representative flow cytometric dot plots showing the proportions of eosinophils, macrophages, neutrophils and monocytes in lung tissue. (E) Percentages of inflammatory cells among lung CD45^+^ leukocytes quantified from panel (D). (F) Methacholine‐induced airway resistance. (G) Serum IgE. (H‐L) IL‐4 (H), IL‐5 (I), IL‐13 (J), IFN‐γ (K) and IL‐17A (L) in lung homogenates. (M) Representative flow cytometric dot plots and quantification of CD25^+^Foxp3^+^ Tregs in spleen. (N) Representative H&E and PAS staining of lung sections (left) and blinded histology scores (right). Scale bars = 50 µm. Data are presented as mean ± SD. *n* = 6 per group. Statistical significance was determined by one‐way ANOVA with Tukey's post hoc test, except for panel (F), in which statistical significance represents the comparison between the OVA and OVA+GUANKE groups at the indicated methacholine concentrations and was determined by two‐way ANOVA followed by Sidak's multiple comparisons test. ^*^ *p* < 0.05, ^**^ *p* < 0.01, ^***^ *p* < 0.001. n.s., not significant.

The OVA‐induced mouse asthma model was established as described with modifications [20, 21]. Briefly, mice were sensitized via intraperitoneal injection with a mixture of 100 µg OVA (purity > 98%, Sigma, USA) and 4 mg alum (Sigma, USA) on days 0 and 7, and subsequently challenged with 3% OVA aerosol for 30 min on days 14 to 16 as shown in Figure 1A. PBS was used as the control for OVA sensitization and challenge. All mice were euthanized 24 h after the final challenge.

To investigate the effect of GUANKE, mice were administered 1 × 10^9^ CFU GUANKE in 200 µL of sterile anaerobic PBS by daily gavage for 16 days. The control group was given an equal volume of PBS by gavage.

For the therapeutic model, mice were sensitized and challenged with OVA as described above. Following the final aerosol challenge on day 16, mice received 1×10^9^ CFU GUANKE in 200 µL of sterile anaerobic PBS by daily gavage for 7 consecutive days. Control groups received an equal volume of PBS. All mice in the therapeutic cohort were euthanized 24 h after the final gavage treatment.

For the IAA efficacy assay in allergic airway inflammation (AAI) mice, mice were treated with 20 mg/kg of IAA (purity > 99%, LABLEAD, China) [22] by daily gavage for 16 days. The control group was given an equal volume of diluted DMSO by gavage.

### 2.5 Antibiotic Treatment and Fecal Microbial Transfer (FMT)

For intestinal microbiota depletion, mice were treated with antibiotic cocktails (ampicillin, 100 mg/kg; metronidazole, 100 mg/kg; vancomycin, 50 mg/kg; neomycin, 100 mg/kg) by daily gavage for 7 days and were then used as recipients of FMT [23]. Following antibiotic treatment, mice were given standard sterile drinking water to ensure antibiotic washout prior to FMT.

For FMT experiments, the procedure was performed as described previously with modifications [24, 25]. Briefly, fecal pellets were collected fresh on day 16 from AAI mice administered by GUANKE or PBS control (donor mice). Pellets were resuspended in sterile PBS (100 mg feces/mL), followed by centrifugation at 1000 × g for 5 min to remove impurity. The supernatant was further centrifuged at 8000 × g for 10 min. The collected samples were resuspended in PBS with 10% (v/v) glycerol and frozen at ‐80°C. Abx‐treated mice (recipient mice) were gavaged with 20 mg of pooled fecal suspension in 100 µL of PBS on days 12–16 during the preparation of the AAI model.

### 2.6 Bmdc Generation and Adoptive Transfer

BMDCs were prepared as described with minor modifications [26]. Bone marrow cells were flushed from the femurs and tibias using sterile PBS and cultured with recombinant mouse GM‐CSF (20 ng/mL) (PeproTech, USA) and mouse IL‐4 (10 ng/mL) (PeproTech, USA) in complete RPMI 1640 medium with 100 U/mL penicillin, 100 µg/mL streptomycin (all from Solarbio, China) and 10% FBS (Corning, USA). Medium was replaced on day 3, and 50% of the medium was replaced on day 5. On day 7, cells were pulsed with OVA (200 µg/mL) or PBS for 24 h. On day 8, medium was supplemented with IAA (250 µM, 500 µM, and 1 mM) or vehicle for 24 h. On day 9, low‐adherent CD11c^+^ cells were sorted using anti‐mouse CD11c magnetic beads (Biolegend, USA).

To address whether the effect of IAA on AAI was associated with DCs, OVA‐IAA‐primed CD11c^+^ BMDCs (1 × 10^6^ cells/mouse) were transferred to recipient mice 4 h before the first challenge timepoint of the allergic asthma murine model on day 14. All recipient mice then received daily 3% OVA aerosol challenge on days 14, 15, and 16.

To evaluate whether Notch signaling contributes to the effects of IAA on AAI, BMDCs (1 × 10^6^ cells/mouse) were incubated after being pulsed with OVA for 24 h and IAA (1 mM) in combination with Jagged1 (2 µg/mL) (MedChemExpress, USA) for 24 h, after which time they were transplanted into WT mice on day 14. The mice were challenged with OVA on days 14–16.

To evaluate the involvement of JAK3‐STAT3 signaling in the effect of IAA, OVA‐pulsed BMDCs were treated with IAA in the presence or absence of the STAT3 agonist colivelin (1 µM, 24 h; MedChemExpress, USA) [27, 28].

### 2.7 In Vitro BMDC and T Cell Co‐Culture

OVA‐IAA‐primed BMDCs were treated as described above. On day 9, purified CD11c^+^ BMDCs (5 × 10^5^) were co‐cultured with splenic CD4^+^ T cells (1.5 × 10^6^) magnetically purified from WT mice. Subsequently, the co‐culture supernatant was collected for ELISA analysis. Cells were then stimulated for 6 h with cell activation cocktail (with Brefeldin A) (BioLegend, USA) and analyzed by flow cytometry for expression of CD4, IL‐4, and IFN‐γ.

### 2.8 Histological Analysis and Goblet Cell Evaluation

Lung tissue was rolled, fixed with 4% paraformaldehyde solution for 24 h, and embedded in paraffin wax. The sections of the embedded tissues were stained with Hematoxylin and Eosin stain and Periodic acid‐Schiff stain. The pathological analysis of stained sections was conducted blindly by three independent pathologists. A scale bar of 50 µm is included in the representative images to indicate the scale. All processes were performed according to the manufacturer's instructions. The slices were collected and evaluated in a blinded manner.

The quantitative assessment of lung pathology was conducted using two established scoring systems. For H&E staining, lung inflammation severity was evaluated based on four key pathological parameters: alveolitis, interstitial inflammation, perivascularitis, and peribronchiolitis. Each parameter was independently graded using an ordinal scoring scale ranging from 0 to 4, where 0 represents normal lung histology (absence of detectable lesions) and 4 designates severe pathological changes. A cumulative lung inflammation score was obtained by summing these individual scores. For PAS staining, goblet cell hyperplasia was quantified based on the percentage of PAS‐positive cells within the airway epithelium using a 5‐point grading scale: 0 (<5% PAS‐positive cells); 1 (5–25%); 2 (25–50%); 3 (50–75%); and 4 (>75%).

### 2.9 Flow Cytometry

Single‐cell suspensions were prepared from lung, peripheral blood, bronchoalveolar lavage fluid (BALF). For lung cell isolation, mice were perfused with PBS, and lungs were dissected and digested in RPMI 1640 medium containing 5% FBS, 1 mg/mL collagenase D (Sigma, USA), and 30 µg/mL DNase I (Sigma, USA) for 30 min at 37 °C [26]. The digested tissue was passed through a 70‐µm cell strainer (Corning, USA). Peripheral blood (100 µL) was collected via cardiac puncture into heparinized tubes [29]. For BALF collection, the trachea was cannulated, and the lungs were lavaged three times with 1 mL of PBS. BALF cells were collected by centrifugation at 500 × g for 5 min. For splenocyte preparation, spleens were mechanically dissociated through 70‐µm strainers into PBS containing 2% FBS. All final single‐cell suspensions were resuspended in FACS buffer for downstream analysis.

Each single‐cell suspension was first incubated with an anti‐CD16/CD32 antibody to block Fc receptors. For surface staining, the following antibodies were used for flow cytometry: anti‐CD45‐AF700, anti‐CD4‐FITC, anti‐SiglecF‐BV421, anti‐Ly6G‐APC/Cy7, anti‐Ly6C‐Percp/Cy5.5, anti‐F4/80‐FITC, anti‐CD11b‐APC, anti‐CD11c‐FITC, anti‐IA‐IE‐BV605, anti‐CD103‐APC, anti‐CD86‐PE, anti‐CX3CR1‐BV421, anti‐CD25‐PE and anti‐CCR2‐PE/Cy7 (all from BioLegend, USA) for 30 min at 4°C in the dark. For intracellular staining, cells were fixed and permeabilized using the Cyto‐Fast Fix/Perm Buffer Set (BioLegend, USA) and stained with anti‐IL‐4‐PE and anti‐IFN‐γ‐APC in the dark at 4°C for 30 min. For nuclear transcription factor detection, cells were processed using the True‐Nuclear Transcription Factor Buffer Set (BioLegend, USA) followed by incubation with anti‐FOXP3‐AF647 antibody. Data were acquired on a BD FACS Celesta flow cytometer and analyzed using FlowJo software (BD Biosciences, USA). The detailed gating strategies for dendritic cells and other immune cell populations are presented in Figure S1.

### 2.10 RT‐qPCR

Peripheral blood was collected via cardiac puncture into EDTA‐coated tubes to prevent coagulation. For RNA extraction, 250 µL of whole blood was immediately mixed with 750 µL of RNAiso Plus (TAKARA, Japan) and homogenized by pipetting. Total RNA was then extracted following the manufacturer's instructions. The concentration and purity of RNA were determined using a NanoDrop spectrophotometer. Subsequently, 1 µg of total RNA was reverse transcribed into cDNA using HiScript IV All‐in‐One Ultra RT SuperMix (Vazyme Biotech Co., Ltd.). RT‐qPCR analysis was performed using ChamQ Blue Universal SYBR qPCR Master Mix (Vazyme Biotech Co., Ltd., China) on LineGene 9600 Fluorescence Quantitative Detection System (Bioer Technology, China). The results were analyzed using the 2^−ΔΔCt^ method and standardized with β‐actin. All specific RT‐qPCR primers are listed in Table S2.

### 2.11 Western Blot

The lung tissues and BMDCs were lysed with RIPA lysis buffer (Solarbio, China) containing a 1% protease and phosphatase inhibitor cocktail to obtain protein samples. The total protein concentration was quantified using a bicinchoninic acid (BCA) protein assay kit (Thermo Scientific, USA). Proteins were separated with SDS‐PAGE and transferred to the nitrocellulose filter membrane (Cytiva, USA). The membranes were blocked with 5% skim milk before being incubated with the primary antibodies overnight. Primary antibodies used were anti‐cleaved Notch4 intracellular domain (NICD4) (sc‐393893, Santa Cruz, USA), p‐STAT3 (9145S, Cell signaling technology, USA), STAT3 (9139S, Cell signaling technology, USA) and anti‐α‐TUBULIN (11224‐1‐AP, Proteintech, USA). The membranes were washed and then incubated with HRP‐labeled secondary antibodies (Lablead, China) at room temperature, followed by signal detection with an ECL kit (Biosharp, China). Image J software was used to detect protein signal levels.

### 2.12 ElisaElisa

Lung homogenate was prepared by dissociating whole lungs in 1 mL PBS containing protease and phosphatase inhibitor cocktail. Suspensions were passed through a 40 µm cell strainer and clarified by centrifugation. Supernatant was used for detecting cytokines by ELISA [30]. Serum IgE and cytokines IL‐4, IL‐5, IL‐13, IFN‐γ, and IL‐17A in the lung or in the cell supernatant were measured using quantitative ELISA kits (Invitrogen, USA) according to the manufacturer's instructions.

### 2.13 Airway Hyperresponsiveness (AHR) Assessment

AHR was invasively assessed using the flexiVent FX system (SCIREQ, Canada). After deep anesthesia with an intraperitoneal injection of pentobarbital sodium (150 mg/kg), mice were subjected to tracheotomy and cannulated with an 18‐gauge stainless steel tube. Lung resistance was then recorded in response to escalating doses of methacholine (MedChemExpress, USA).

### 2.14 16S RRNA Amplicon Sequencing

16S rRNA amplicon sequencing was performed at Majorbio Bio‐pharm Biotechnology Co., Ltd. (Shanghai, China) on fecal samples. Bacterial community analysis involved amplification of the 16S rRNA gene using universal primers 27F (5’‐AGRGTTYGATYMTGGCTCAG‐3’) and 1492R (5’‐RGYTACCTTGTTACGACTT‐3’). PCR products were electrophoresed, purified with AMPure PB beads (Pacific Biosciences), and quantified via Qubit 4.0 fluorometer (Thermo Fisher Scientific). Equimolar purified amplicons were pooled, and libraries were constructed with the SMRTbell Prep Kit 3.0 (Pacific Biosciences) per manufacturer's protocol. High‐fidelity (HiFi) reads were generated through circular consensus sequencing (SMRT Link v11.0). Amplicon sequence variants (ASVs) were taxonomically classified using QIIME2's naïve Bayes consensus classifier against the SILVA 138 database. Microbial diversity was assessed by calculating α‐diversity (Shannon index) and β‐diversity via principal coordinate analysis (PCoA) based on weighted UniFrac distances. Differentially abundant bacterial genera were identified by linear discriminant analysis effect size (LEfSe) (LDA score >2, *P*<0.05).

For species‐level differential analysis, relative abundance profiles assigned at the species level were compared across the Control, OVA, and OVA+GUANKE groups using a multi‐group differential testing workflow. Species were ranked according to the raw *p* values obtained from the overall comparison, while the corresponding multiple‐testing‐adjusted *p* values were recorded in parallel. The 10 species with the smallest raw *p* values were defined as the top differentially altered species and were selected for visualization in Figure S2. For plotting, the relative abundance of each selected species in individual samples was extracted from the species‐level abundance table.

### 2.15 High Throughput Targeted Metabolomics

Samples, including intestinal content were homogenized in 20 µL deionized water and 120 µL lysis buffer. The mixture was diluted with 90 µL of pre‐cooled diluent, vortexed and centrifuged prior to LC‐MS/MS analysis. The LC‐MS/MS analysis of the sample was carried out at BGI Genomics Co., Ltd. (Shenzhen, China). Chromatographic separation was performed with mobile phase A (0.1% formic acid in water) and B (acetonitrile:isopropanol = 7:3). Mass spectrometry was performed on a Q Exactive instrument (Thermo Fisher Scientific) in switching ESI mode. The full scan range was 70‒1050 m/z with a resolution of 70000, and the automatic gain control (AGC) target for MS acquisitions was set to 3 × 10^6^ with a maximum ion injection time of 100 ms. Metabolite identification and quantification were performed using Skyline (v21.1.0.146) with monoisotopic mass tolerance (0.6 Da) and mass range (50–1500 Da).

### 2.16 Targeted Serum Tryptophan Metabolomics

Targeted serum tryptophan metabolomic analysis was performed by Shanghai Majorbio Bio‐pharm Technology Co., Ltd. Mixed calibration standards were prepared for 33 tryptophan‐related metabolites, and indole‐2H5‐L‐tryptophan (Trp‐d5) was used as the internal standard. Briefly, 100 µL of serum was mixed with 10 µL of internal standard solution and 390 µL of methanol. Samples were vortexed for 30 s, sonicated at 40 kHz and 5°C for 30 min, incubated at −20°C for 30 min, and centrifuged at 13000 × g and 4°C for 15 min. Subsequently, 350 µL of the supernatant was evaporated under nitrogen, reconstituted in 140 µL of 1% acetonitrile containing 0.1% formic acid, and centrifuged again after vortexing and sonication.

LC‐MS/MS analysis was performed using a Nexera Series LC‐40 system coupled to a QTRAP 6500+ mass spectrometer. Metabolites were separated on an ACQUITY UPLC HSS T3 column (2.1 × 150 mm, 1.8 µm) at 40°C using water and acetonitrile, both containing 0.1% formic acid, at a flow rate of 0.35 mL/min. The proportion of acetonitrile was increased from 5% to 30% over 0–4 min and to 100% at 7 min, maintained until 8.5 min, and returned to 5% at 9 min for equilibration until 12 min. Detection was performed in positive and negative electrospray ionization modes using multiple reaction monitoring.

Chromatographic peaks were integrated using SCIEX OS software and manually inspected. Metabolite concentrations were calculated from analyte‐to‐internal‐standard peak‐area ratios using linear calibration curves. Quality‐control samples were injected every 5–10 samples, with relative standard deviations below 15%. Data are presented as median (interquartile range). Group differences were evaluated using two‐sided Mann‐Whitney *U* tests, with *p* values adjusted using the Benjamini‐Hochberg method. An FDR‐adjusted *p* value < 0.05 was considered statistically significant.

### 2.17 Transcriptomics Analysis

Purified CD11c^+^ BMDCs were pulsed with OVA (200 µg/mL) and treated with IAA (1 mM) for 24 h. Cells were collected and total RNA was extracted using RNAiso Plus (TAKARA) per manufacturer's protocol. RNA sequencing services (including purification, cDNA library construction, and Illumina sequencing) were provided by Majorbio Bio‐pharm Biotechnology Co., Ltd. Specifically, 1 µg total RNA was used to prepare stranded mRNA libraries (Illumina Stranded mRNA Prep, Ligation) through fragmentation and adapter ligation. cDNA fragments (300 bp) were size‐selected via 2% Low Range Ultra Agarose electrophoresis and amplified with Phusion DNA polymerase (NEB) for 15 cycles. Libraries were quantified by Qubit 4.0 and sequenced on NovaSeq X Plus (PE150; NovaSeq Reagent Kit). Raw paired‐end reads were quality‐trimmed using fastp (default parameters). Clean reads were aligned to the reference genome in orientation‐aware mode with HISAT2, then assembled via reference‐guided StringTie. Gene expression was quantified as TPM (transcripts per million) using RSEM. Differential expression analysis between groups was performed with DESeq2. Kyoto Encyclopedia of Genes and Genomes (KEGG) pathway enrichment was assessed using Goatools and SciPy.

### 2.18 Chromatin Immunoprecipitation‐qPCR

Chromatin immunoprecipitation followed by quantitative PCR (ChIP‐qPCR) was performed using Magna ChIP A/G Kit (Millipore; 17–10085) according to the manufacturer's instructions. Briefly, chromatin from Control, OVA‐pulsed, and OVA+IAA‐treated BMDCs was cross‐linked, sheared, and immunoprecipitated with an anti‐STAT3 antibody (9139S, Cell Signaling Technology, USA) or normal rabbit IgG as a negative immunoprecipitation control. Purified DNA was analyzed by qPCR using two primer sets within the mouse *Jag1* promoter encompassing predicted STAT3‐binding motifs. STAT3 enrichment was normalized to the corresponding input chromatin and expressed as a percentage of input. The primer sequences used for ChIP‐qPCR are provided in Table S3.

### 2.19 Lactate Dehydrogenase (LDH) Cytotoxicity Assay

According to the manufacturer's protocol, cytotoxicity was quantified by measuring the released LDH activity using the Cytotoxicity Detection Kit (LDH) (Promega, USA). Briefly, 50 µL culture media were collected after 400 × g centrifugation for 5 min. 50 µL of prepared LDH test working solution was added into each sample, which was incubated at room temperature (about 25°C) in the dark for 30 min. Subsequently, the absorbance was measured at 490 nm using a microplate reader. The LDH relative release amount was calculated using the following formula: LDH relative release amount (%) = (OD [treated sample]—OD [control sample]) / (OD [maximum enzyme activity of cells]—OD [control sample]) × 100%.

### 2.20 Statistical Analysis

Data are presented as mean ± SD unless otherwise indicated. Demographic and clinical characteristics in Table S1 are presented as median (interquartile range, IQR) for non‐normally distributed continuous variables and as numbers for categorical variables. Statistical analyses were performed using GraphPad Prism (v 9.0). Normality and homogeneity of variance were assessed using the Shapiro‐Wilk and Brown‐Forsythe tests, respectively. Comparisons between two independent groups were performed using an unpaired two‐tailed Student's *t*‐test for normally distributed data or a two‐sided Mann‐Whitney *U* test for non‐normally distributed data. Categorical variables were compared using Fisher's exact test. Comparisons among three or more groups were performed using one‐way ANOVA followed by Tukey's multiple comparisons test. Experiments involving two independent factors were analyzed using two‐way ANOVA followed by Tukey's multiple comparisons test. Methacholine dose‐response data were analyzed using two‐way ANOVA followed by Sidak's multiple comparisons test. *p* < 0.05 was considered statistically significant.

## 3 Results

### 3.1 Guanke Administration Relieves OVA‐Induced Allergic Asthma

To validate the immunomodulatory role of GUANKE in allergic asthma, we established an OVA‐induced murine model in which mice received oral administration of GUANKE or PBS as a control (Figure 1A). Compared to the control group, OVA treatment elicited marked inflammatory cell infiltration in both the airways and lung parenchyma. In the BALF, this was evidenced by a substantial increase in total cell counts (Figure 1B), with differential analysis revealing specific accumulation of eosinophils, macrophages, and neutrophils (Figure 1C). Consistent with these airway findings, flow cytometric analysis of lung single‐cell suspensions demonstrated a parallel increase in these tissue‐resident inflammatory cells (Figure 1D,E). Notably, GUANKE treatment significantly attenuated this pathological cellular infiltration in both compartments (Figure 1B–E). Invasive assessment of airway physiology further showed that OVA challenge markedly increased methacholine‐induced airway resistance, whereas GUANKE administration significantly reduced this response, indicating amelioration of airway hyperresponsiveness (Figure 1F).

Furthermore, GUANKE‐treated mice exhibited significantly lower serum IgE levels and suppressed production of Th2‐associated cytokines (IL‐4, IL‐5, and IL‐13) compared to the AAI model group (Figure 1G–J). Despite these potent anti‐Th2 effects, GUANKE treatment failed to reverse the OVA‐induced downregulation of the Th1 cytokine IFN‐γ or the elevation of the Th17 cytokine IL‐17A (Figure 1K,L), indicating a Th2‐biased mechanism of action. Regarding immune homeostasis, flow cytometric analysis revealed that while OVA treatment significantly reduced the CD4^+^CD25^+^Foxp3^+^ Treg population, GUANKE intervention partially restored this critical immunosuppressive subset (Figure 1M). Consistently, histological analyses using H&E and PAS staining visually confirmed the effective reduction of pulmonary eosinophilic infiltration and significant inhibition of airway mucus hypersecretion following GUANKE treatment (Figure 1N).

Given these prophylactic benefits, we further evaluated the therapeutic potential of GUANKE in established allergic asthma by administering it for 7 consecutive days immediately after the final OVA challenge (Figure S3A). Therapeutic administration recapitulated the protective effects observed in the prophylactic model, effectively alleviating airway inflammation, as evidenced by reduced pulmonary infiltration of eosinophils, macrophages, and neutrophils (Figure S3B,C). Similarly, therapeutic treatment suppressed Th2 immune responses, including serum IgE and pulmonary IL‐5 levels (Figure S3D,E), without altering the Th1/Th17 cytokine profile (Figure S3F,G). Collectively, these data demonstrate that GUANKE possesses both prophylactic and therapeutic efficacy against AAI, primarily through suppression of Th2‐driven responses and partial restoration of Treg populations.

### 3.2 GUANKE Treatment Reduces OVA‐induced CD11b+ DC Recruitment and Activation

**FIGURE 2:**
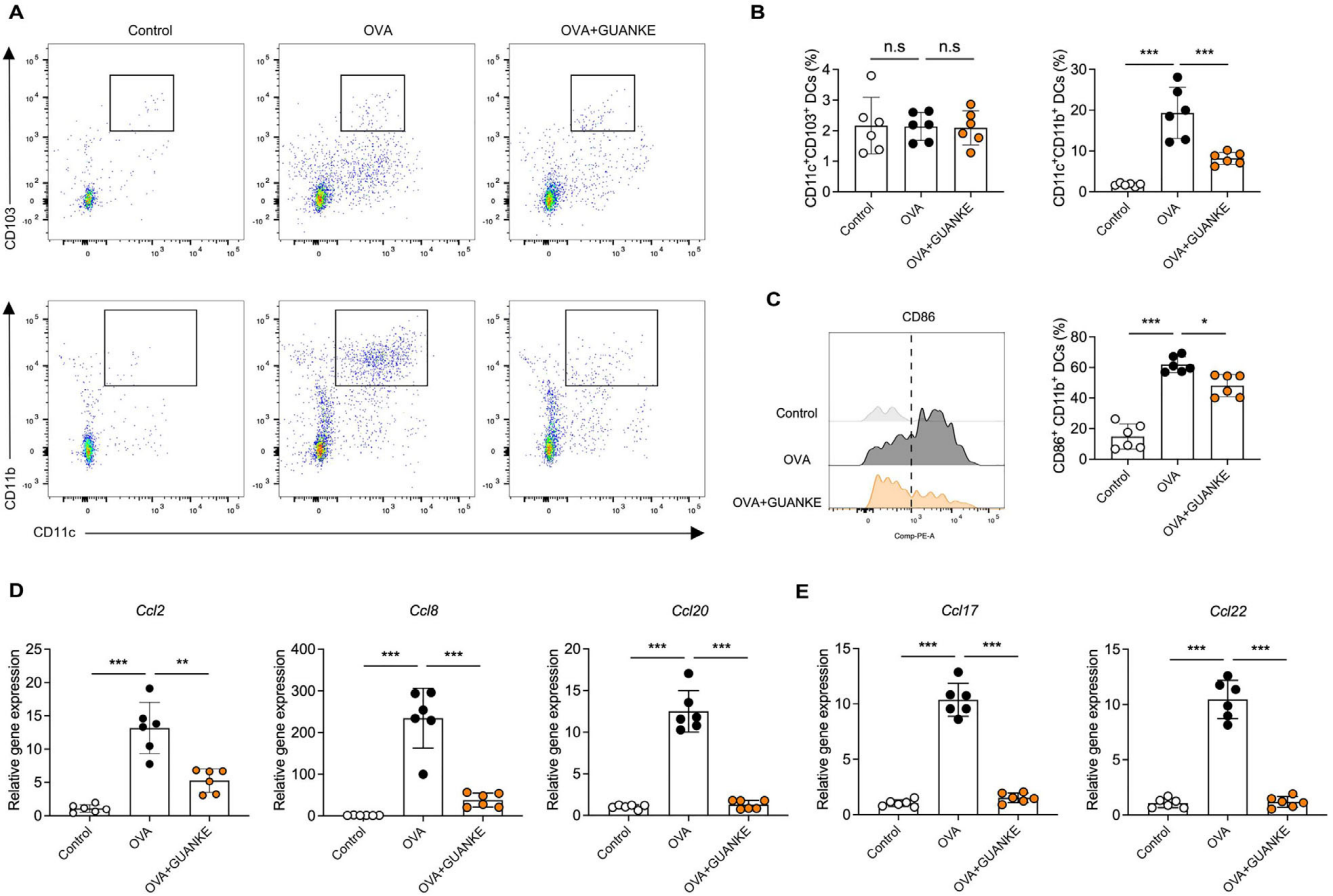
Accumulation and activation of CD11b^+^ DCs in the lung were inhibited following GUANKE treatment. (A) Representative flow cytometric dot plots showing the proportions of CD103^+^ DCs and CD11b^+^ DCs of total DCs in lung tissue. (B) The frequency of CD103^+^ DCs and CD11b^+^ DCs quantified from panel (A). (C) Representative flow cytometry histogram (left) and percentages of CD86^+^ cells among CD11b^+^ DCs (right). (D‐E) mRNA expression of chemokines in the lungs of AAI mice measured by RT‐qPCR. Data are presented as mean ± SD. *n* = 6 per group. Statistical significance was determined by one‐way ANOVA with Tukey's post hoc test. ^*^ *p* < 0.05, ^**^ *p* < 0.01, ^***^ *p* < 0.001. n.s., not significant.

In response to allergen stimulation, lung DCs take up antigens and present them to polarize the Th2 immune response [8, 26]. Subsequent investigations were conducted to determine whether the GUANKE‐induced attenuation of Th2 responses in asthma was associated with alterations in DC recruitment. Flow cytometric analysis revealed that prophylactic GUANKE treatment significantly suppressed OVA‐induced accumulation of CD11b^+^ DCs, while concurrently inducing a modest increase in the proportion of CD103^+^ DC (Figure 2A,B). Beyond reducing cell abundance, GUANKE also impaired the functional maturation of CD11b^+^ DCs, as evidenced by downregulated surface expression of the costimulatory molecule CD86 (Figure 2C). Importantly, this suppressive effect extended beyond disease prevention, as GUANKE administration similarly inhibited the pulmonary expansion of CD11b^+^ DCs in our therapeutic model of established asthma (Figure S3H). The consistency of this effect across both experimental settings suggests that reduced recruitment and activation of CD11b^+^ DCs are important cellular features associated with GUANKE‐mediated protection.

To further elucidate the basis of this reduced recruitment, we examined the expression of DC‐associated chemokines. GUANKE treatment significantly inhibited the pulmonary expression of *Ccl2*, *Ccl8*, and *Ccl20* (Figure 2D), key chemokines secreted by airway epithelial cells that mobilize DC precursors. This downregulation aligns with the observed reduction in CD11b^+^ DC accumulation in vivo. Furthermore, the expression of *Ccl17* and *Ccl22*, chemokines predominantly produced by mature DCs to recruit Th2 cells, was also diminished in GUANKE‐treated mice (Figure 2E). Collectively, these results indicate that the suppression of these specific chemotactic signals contributes to the reduced recruitment and activation of CD11b^+^ DCs following GUANKE treatment.

### 3.3 GUANKE Restores IAA Levels in AAI Mice, and Patients With Asthma Exhibit Reduced Circulating IAA

**FIGURE 3:**
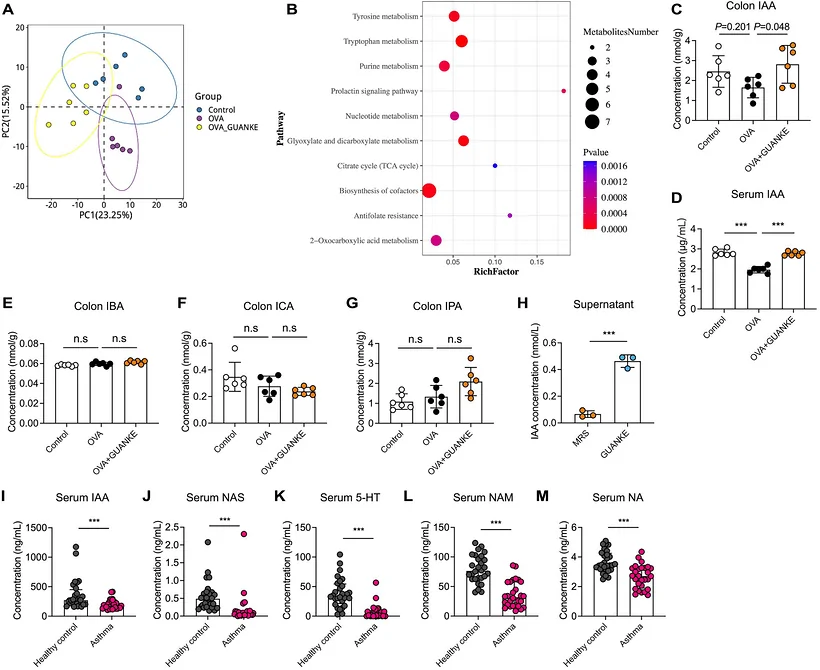
Targeted metabolomic characterization of IAA and related tryptophan metabolites in experimental and clinical asthma. (A) PLS‐DA of intestinal metabolite profiles from the Control, OVA and OVA+GUANKE groups. (B) KEGG analysis of differentially regulated metabolites between the OVA group and the OVA+GUANKE group. (C‐D) Quantification of IAA concentrations in mouse colonic contents (C) and serum (D). (E‐G) Quantification of IBA (E), ICA (F) and IPA (G) concentrations in mouse colonic contents. (H) Quantification of IAA in GUANKE culture supernatants and the MRS medium control. (I‐M) Serum concentrations of IAA (I), NAS (J), 5‐HT (K), NAM (L), and NA (M) in healthy controls and patients with asthma. IAA, indole‐3‐acetic acid; IBA, 3‐indolebutyric acid; ICA, indole‐3‐carboxylic acid; IPA, indole‐3‐propionic acid; NAS, N‐acetylserotonin; 5‐HT, serotonin; NAM, nicotinamide; NA, nicotinic acid. Data for panels C‐H are presented as mean ± SD, whereas data for panels I‐M are presented as median (interquartile range). *n* = 6 per group for panels A‐G, *n* = 3 per group for panel H, and *n* = 28 per group for panels I‐M. Statistical significance was determined using one‐way ANOVA followed by Tukey's multiple comparisons test for panels C‐G and an unpaired two‐tailed Student's *t*‐test for panel H. Panels I‐M were analyzed using two‐sided Mann‐Whitney *U* tests, with *p* values adjusted using the Benjamini‐Hochberg method. For panels I‐M, asterisks denote Benjamini‐Hochberg‐adjusted *P* values. For the remaining quantitative panels, asterisks denote unadjusted *P* values. ^*^ *P* or adjusted *p* < 0.05, ^**^ *P* or adjusted *p* < 0.01, and ^***^ *P* or adjusted *p* < 0.001.

To elucidate the metabolic changes associated with the protective effect of GUANKE, we conducted high‐throughput targeted metabolomic profiling of intestinal contents from the control, OVA, and OVA+GUANKE groups. Partial least‐squares discrimination analysis (PLS‐DA) revealed distinct metabolic profiles among the three groups, with clear separation between the OVA and OVA+GUANKE groups (Figure 3A). KEGG pathway enrichment analysis identified tryptophan metabolism as one of the pathways significantly altered following GUANKE administration (Figure 3B). Within this pathway, colonic IAA levels showed a nonsignificant downward trend in AAI mice compared with the control group but were significantly increased by GUANKE treatment (Figure 3C). Consistently, serum IAA levels were markedly reduced in the AAI group and restored following GUANKE administration (Figure 3D). In contrast, colonic levels of other tryptophan metabolites, including 3‐indolebutyric acid (IBA), indole‐3‐carboxylic acid (ICA), and indole‐3‐propionic acid (IPA), were not significantly altered by either OVA challenge or GUANKE intervention (Figure 3E–G). In vitro analysis further showed that IAA concentrations were significantly higher in GUANKE culture supernatants than in the MRS medium control (Figure 3H). Therapeutic administration of GUANKE similarly reversed the reduction in serum IAA observed in AAI mice (Figure S3I). Collectively, these findings indicate that restoration of colonic and circulating IAA are the consistent metabolic features associated with GUANKE‐mediated protection in AAI.

To evaluate the clinical relevance of the IAA‐related findings, we performed targeted serum tryptophan metabolomic profiling in patients with asthma and healthy controls. Consistent with the reduction observed in AAI mice, serum IAA levels were significantly lower in patients with asthma than in healthy controls (Figure 3I). In addition, several other circulating metabolites, including N‐acetylserotonin (NAS), serotonin (5‐HT), nicotinamide (NAM), and nicotinic acid (NA), were also significantly lower in patients with asthma (Figure 3J–M), indicating broader alterations in circulating tryptophan‐related metabolism. Together, these murine and human data highlight the reduced circulating IAA as a metabolic feature shared by experimental AAI and human asthma.

### 3.4 Iaa Administration Reproduces Key Features of Guanke‐mediated Protection in AAI

**FIGURE 4:**
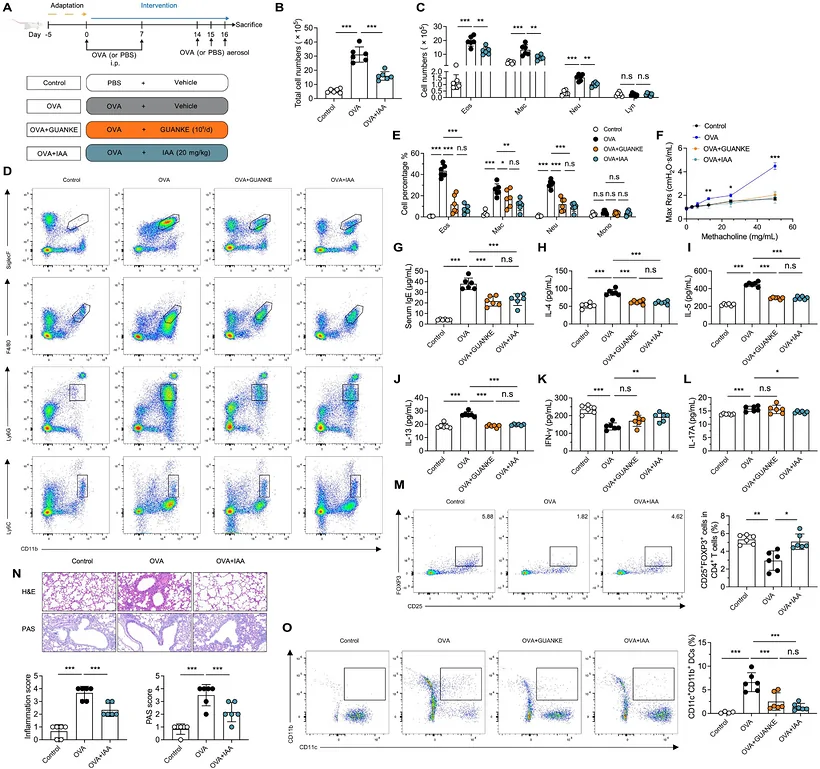
Effects of IAA supplementation on airway inflammation and immune responses in AAI mice. (A) Experimental scheme of the animal experimental design. Mice were administered IAA (20 mg/kg), GUANKE (1×10^9^ CFU), or vehicle by daily gavage for 16 days, sensitized with OVA or PBS via intraperitoneal (i.p.) injection on days 0 and 7, and challenged with OVA or PBS aerosol from day 14 to 16. (B) Total cell counts were determined in the BALF of mice. (C) Number of eosinophils (Eos), macrophages (Mac), neutrophils (Neu), and lymphocytes (Lyn) in BALF. (D) Representative flow cytometric dot plots showing the proportions of eosinophils, macrophages, neutrophils and monocytes in lung tissue from the Control, OVA, OVA+GUANKE, and OVA+IAA groups. (E) Percentages of inflammatory cells among lung CD45^+^ leukocytes quantified from panel (D). (F) Methacholine‐induced airway resistance. (G) Serum IgE. (H‐L) IL‐4 (H), IL‐5 (I), IL‐13 (J), IFN‐γ (K), and IL‐17A (L) in lung homogenates. (M) Representative flow cytometric dot plots and quantification of CD25^+^Foxp3^+^ Tregs in spleen. (N) Representative H&E and PAS staining of lung sections and blinded histology scores. Scale bars = 50 µm.(O) Representative flow cytometric dot plots and statistical analysis of the frequency of CD11b^+^ DCs in lungs. Data are presented as mean ± SD. *n* = 6 per group. Statistical significance was determined by one‐way ANOVA with Tukey's post hoc test except for panel (F), which was analyzed by two‐way ANOVA followed by Sidak's multiple comparisons test. ^*^ *p* < 0.05, ^**^ *p* < 0.01, ^***^ *p* < 0.001. n.s., not significant.

Following the identification of IAA as a metabolite restored by GUANKE treatment, we next evaluated the effect of exogenous IAA administration in the OVA‐induced AAI model (Figure 4A). IAA significantly reduced the OVA‐induced increase in total BALF cell numbers and decreased the accumulation of eosinophils, macrophages, and neutrophils, without significantly affecting lymphocyte numbers (Figure 4B,C). Consistent changes were observed in lung tissue, where IAA reduced the proportions of eosinophils, macrophages, and neutrophils but did not alter the monocyte population (Figure 4D,E). These reductions in airway and pulmonary inflammation were accompanied by attenuation in methacholine‐induced airway resistance, indicating that the effects of IAA extended to improvement of airway function (Figure 4F).

Similar to GUANKE treatment, IAA administration suppressed the elevation of serum IgE and pulmonary Th2 cytokines (Figure 4G–J). However, IAA also restored the OVA‐induced reduction in IFN‐γ and suppressed the elevation of IL‐17A, neither of which was significantly altered by GUANKE treatment (Figure 4K,L). In addition, IAA partially restored the splenic CD4^+^CD25^+^Foxp3^+^ Treg population (Figure 4M). Histological analysis showed reduction in pulmonary inflammatory infiltration and airway goblet cell hyperplasia following IAA administration (Figure 4N). Notably, IAA also reproduced the inhibitory effect of GUANKE on the accumulation of pulmonary CD11b^+^ DCs (Figure 4O). Together, these findings indicate that IAA largely reproduces the protective profile of GUANKE while exerting additional regulatory effects on Th1 and Th17 responses.

### 3.5 GUANKE Reshapes the Gut Microbiota in Association With Microbiota‐Dependent Restoration of Endogenous IAA

**FIGURE 5:**
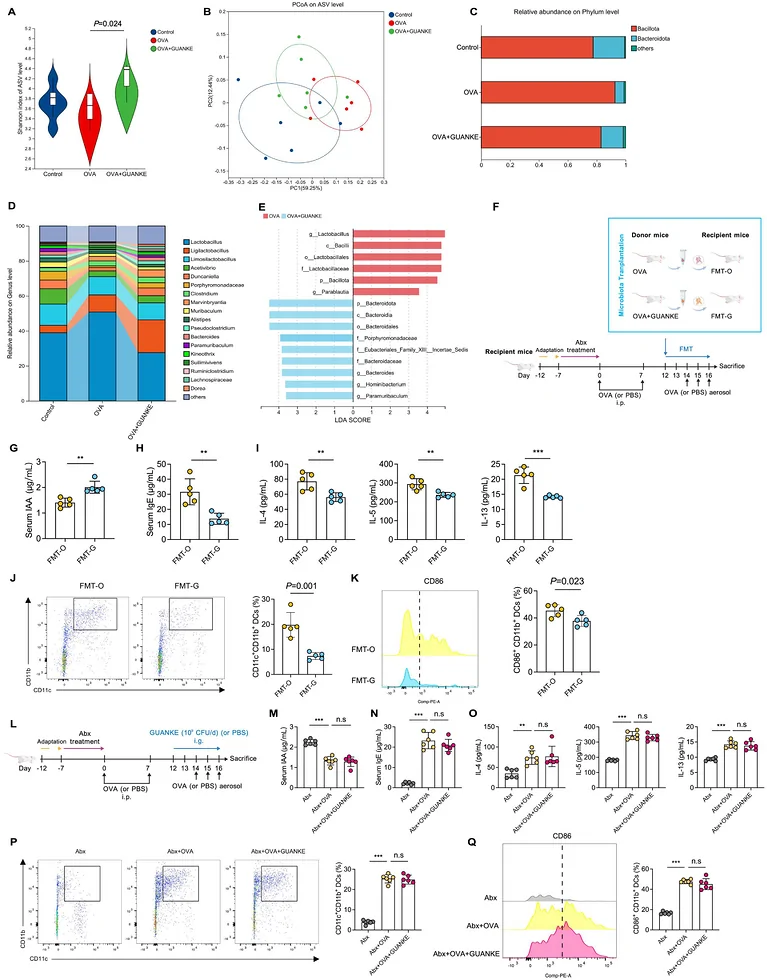
GUANKE reshaped the gut microbiota and was associated with microbiota‐dependent restoration of circulating IAA and attenuation of allergic asthma. (A) Alpha diversity of gut microbiota measured by the Shannon index at the ASV level (*n* = 6). (B) PCoA based on weighted UniFrac distance showing distinct clustering of gut microbiota communities (*n* = 6). (C) Relative abundance of gut microbiota at the phylum level. (D) Relative abundance of dominant taxa at the genus level. LEfSe analysis identifying differentially enriched bacterial taxa between the OVA and OVA+GUANKE groups (LDA score > 2.0). (E, F) Experimental scheme of the FMT experiment. Recipient mice were pretreated with a broad‐spectrum antibiotic cocktail (ampicillin, vancomycin, neomycin, and metronidazole) for 7 days, followed by OVA sensitization and challenge. Donor fecal microbiota from OVA or OVA+GUANKE mice was administered by oral gavage from days 12–16. Serum IAA levels in FMT recipients (*n* = 5). (G, H) Serum IgE in FMT recipients (*n* = 5). (I) IL‐4, IL‐5 and IL‐13 in lung homogenates of FMT recipients (*n* = 5). (J) Representative flow cytometric dot plots and the statistical analysis of the frequency of CD11b^+^ DCs in lung tissue of FMT recipient mice (*n* = 5). Representative flow cytometry histogram (left) and percentages of CD86^+^ cells among CD11b^+^ DCs (right) in FMT recipient mice (*n* = 5). (K, L) Experimental scheme of the direct GUANKE supplementation experiment in microbiota‐depleted mice. Abx‐pretreated mice were sensitized and challenged with OVA and received oral gavage of GUANKE (1×10^9^ CFU) or PBS from days 12–16. (M) Serum IAA (*n* = 6). (N) Serum IgE (*n* = 6). (O) IL‐4, IL‐5 and IL‐13 in lung homogenates (*n* = 6). (P) Representative flow cytometric dot plots and the statistical analysis of the frequency of CD11b^+^ DCs in lung tissue (*n* = 6). (Q) Representative flow cytometry histogram (left) and percentages of CD86^+^ cells among CD11b^+^ DCs (right) (*n* = 6). Data are presented as mean ± SD. Statistical significance was determined by one‐way ANOVA followed by Tukey's multiple comparisons test for panels A and M‐Q and by an unpaired two‐tailed Student's *t*‐test for panels G‐K. LEfSe analysis in panel E used an LDA score > 2.0 and *p* < 0.05. ^*^ *p* < 0.05, ^**^ *p* < 0.01, ^***^ *p* < 0.001. n.s., not significant.

To investigate the involvement of altered microbial population in GUANKE‐associated IAA restoration, we performed full‐length 16S rRNA sequencing of fecal samples from AAI mice. GUANKE administration increased microbial α‐diversity, as indicated by the Shannon index (*p* = 0.024, Figure 5A), and shifted overall community structure (Figure 5B). At the phylum level, GUANKE treatment altered the OVA‐associated relative abundance pattern of *Bacillota* and *Bacteroidota* [31] (Figure 5C). Genus‐level profiling further revealed distinct changes in the dominant microbial taxa (Figure 5D), while LEfSe analysis identified enrichment of the genus *Bacteroides* and members of the order Eubacteriales in GUANKE‐treated mice compared with the OVA group (Figure 5E).

Among the top 10 differentially abundant species, four species belonging to the *Bacteroides* genus were enriched after GUANKE treatment, including *Bacteroides uniformis*, *Bacteroides faecichinchillae*, *Bacteroides caecimuris*, and *Bacteroides acidifaciens* (Figure S2). Because several members of the *Bacteroides* genus have been implicated in tryptophan metabolism [32, 33], the parallel enrichment of these taxa and restoration of circulating IAA highlight them as candidate species associated with the GUANKE‐induced metabolic changes.

To determine whether the GUANKE‐modified microbial community could transfer the IAA‐restoring and protective phenotype, we performed fecal microbiota transplantation (FMT) into antibiotic (Abx)‐pretreated recipient mice (Figure 5F). Compared with recipients of fecal microbiota from OVA‐treated donors (FMT‐O), recipients of microbiota from GUANKE‐treated donors (FMT‐G) exhibited higher serum IAA levels (Figure 5G). This metabolic recovery was accompanied by reduced serum IgE and pulmonary Th2 cytokine levels (Figure 5H,I), together with decreased recruitment and activation of pulmonary CD11b^+^ DCs (Figure 5J,K).

We next examined whether direct GUANKE administration could restore IAA after Abx‐mediated depletion of the resident microbiota (Figure 5L). In contrast to the FMT results, direct GUANKE supplementation did not restore serum IAA (Figure 5M), reduce serum IgE or pulmonary IL‐4, IL‐5, and IL‐13 levels (Figure 5N,O), or inhibit pulmonary CD11b^+^ DC accumulation and activation (Figure 5P,Q). Collectively, the contrasting outcomes of FMT from GUANKE‐treated donors and direct GUANKE administration after Abx pretreatment support a microbiota‐dependent contribution to the restoration of circulating IAA and the associated protection against AAI.

### 3.6 IAA Modulates DC Function and Attenuates Th2‐Dominated AAI

**FIGURE 6:**
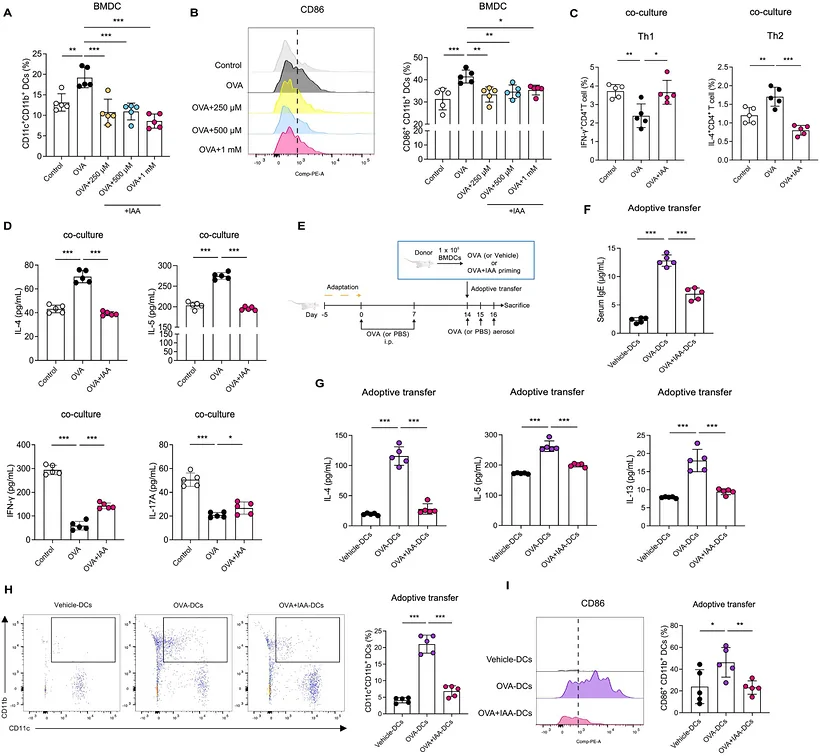
IAA‐conditioned BMDCs exhibited reduced Th2‐polarizing activity in vitro and attenuated Th2 inflammation after adoptive transfer. (A) The frequency of CD11c^+^CD11b^+^ BMDCs among CD11c^+^ BMDCs treated with varying concentrations of IAA or vehicle. (B) Representative flow cytometry histogram (left) and percentages of CD86^+^ cells among CD11b^+^ DCs (right). (C) The frequency of CD4^+^IFN‐γ^+^ Th1 cells and CD4^+^IL‐4^+^ Th2 cells among CD4+T cells co‐cultured with vehicle‐, OVA‐, or OVA+IAA‐treated BMDCs. (D) IL‐4, IL‐5, IFN‐γ and IL‐17A in co‐culture supernatants. (E) Experimental scheme of adoptive transfer. BMDCs pulsed with OVA in the presence or absence of IAA and enriched by magnetic cell sorting. CD11c^+^ BMDCs (1×10^6^) were adoptively transferred into recipient mice 4 h before OVA challenge on day 14, followed by sequential challenge with OVA from days 14–16. Mice were sacrificed for analysis on day 17. Serum IgE levels in recipient mice. (F, G) IL‐4, IL‐5 and IL‐13 in lung homogenates of recipient mice. (H) Representative flow cytometric dot plots and the statistical analysis of the frequency of CD11b^+^ DCs in lung tissue of recipient mice. (I) Representative flow cytometry histogram (left) and percentages of CD86^+^ cells among CD11b^+^ DCs (right). Data are presented as mean ± SD. *n* = 5 per group. Statistical significance was determined by one‐way ANOVA with Tukey's post hoc test. ^*^ *p* < 0.05, ^**^ *p* < 0.01, ^***^ *p* < 0.001. n.s., not significant.

To further determine whether IAA suppresses the accumulation and activation of CD11b^+^ DCs in vitro, BMDCs were pulsed with OVA for 24 h, and subsequently treated with IAA at different concentrations. IAA treatment (250 µM, 500 µM, and 1 mM) did not induce significant cytotoxicity compared to the control (Figure S4), confirming that the subsequent functional effects were not attributable to reduced cell viability. As shown in Figure 6AB, various concentrations of IAA inhibited OVA‐induced CD11b^+^ DC accumulation and activation, with the strongest effect observed at 1 mM. These in vitro results are consistent with the reduced CD11b^+^ DC accumulation and activation observed in the lungs of IAA‐treated mice, indicating that IAA acts directly on DCs.

We next performed a co‐culture assay of splenic CD4^+^ T cells with BMDCs to assess the impact of IAA on DC‐mediated T cell polarization. Flow cytometry analysis revealed that OVA‐pulsed BMDCs treated with IAA led to a significant reduction in IL‐4‐producing Th2 cells and an increase in IFN‐γ‐producing Th1 cells, compared to vehicle‐treated, OVA‐pulsed BMDCs (Figure 6C). In line with the flow cytometry data, cytokine profiling confirmed decreased levels of Th2‐associated cytokines (IL‐4 and IL‐5), and further revealed elevated levels of Th1‐ and Th17‐associated cytokines in the co‐culture supernatants (Figure 6D). These results indicate that IAA reprograms BMDCs toward a phenotype that attenuates Th2 polarization and promotes Th1/Th17 differentiation.

To evaluate whether IAA alleviates allergic asthma through DC‐mediated immunoregulation in vivo, we adoptively transferred OVA‐pulsed BMDCs that had been stimulated with or without IAA into OVA‐challenged mice (Figure 6E). Mice receiving IAA‐conditioned BMDCs exhibited reduced serum IgE levels and lower concentrations of Th2 cytokines in the lungs (Figure 6F,G), as well as lower frequencies of CD11b^+^ DCs and CD86 expression on CD11b^+^ DCs (Figure 6H,I). These findings demonstrate that IAA modulates DC function to suppress AAI, underscoring its role in DC‐driven immune reprogramming.

### 3.7 IAA Reduces CX3CR1^+^Ly6C^−^ Precursors and Pulmonary CD11b^+^ DC Accumulation After CD11c‐Specific AhR Deletion

**FIGURE 7:**
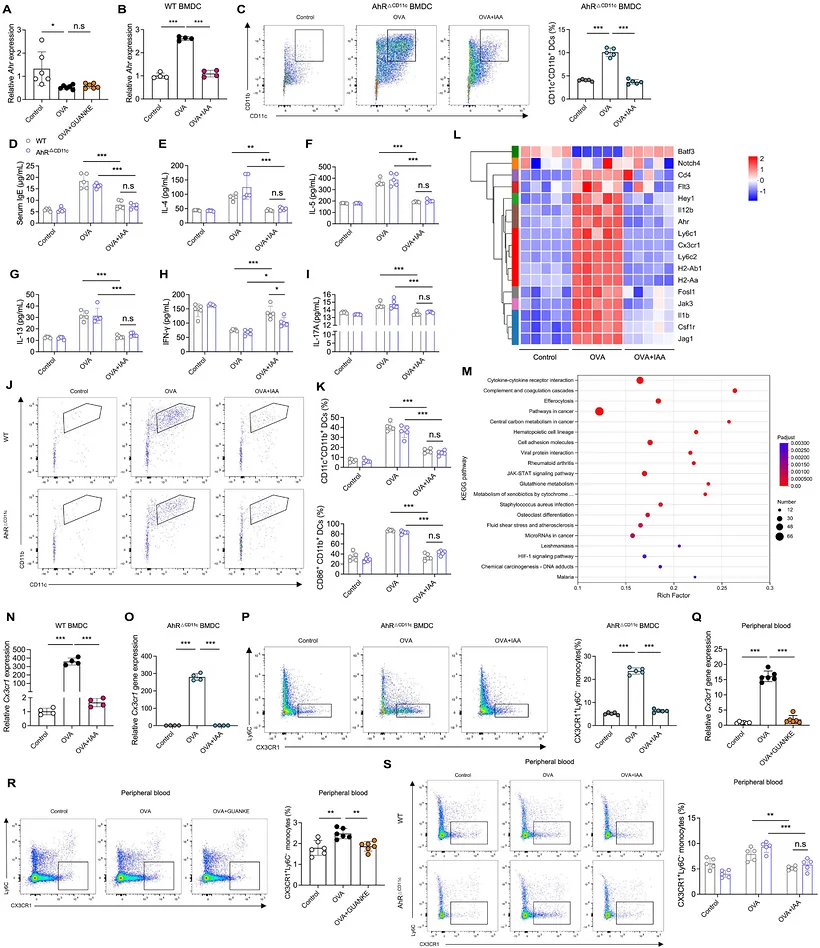
IAA limited the expansion of circulating CX3CR1^+^Ly6C^−^ precursor cells after CD11c‐specific AhR deletion. (A) mRNA expression of *Ahr* in the lungs of mice treated with or without GUANKE (*n* = 6). (B) mRNA expression of *Ahr* in the WT BMDC treated with OVA in the presence or absence of IAA (*n* = 4). (C) Representative flow cytometric dot plots and the statistical analysis of the frequency of CD11b^+^ DCs in AhR^△CD11c^ BMDCs (*n* = 5). (D) Serum IgE (*n* = 5). (E‐I) IL‐4 (E), IL‐5 (F), IL‐13 (G), IFN‐γ (H), and IL‐17A (I) in lung homogenates (*n* = 5). (J) Representative flow cytometric dot plots showing the proportions of CD11b^+^ DCs among total DCs in lung tissue of WT and AhR^△CD11c^ mice (*n* = 5). (K) The frequency of CD11b^+^ DCs and CD86^+^CD11b^+^DCs in lung tissue of WT and AhR^△CD11c^ mice (*n* = 5). (L) Heatmap showing the expression profiles of 17 genes associated with DC differentiation, immune responses, and the Notch/AhR signaling pathway. (M) KEGG pathway enrichment analysis of differentially expressed genes between OVA‐pulsed BMDCs and OVA‐IAA‐pulsed BMDCs. (N‐O) mRNA expression of *Cx3cr1* in WT BMDCs (N) and in AhR^△CD11c^ BMDCs (O) (*n* = 4). (P) Representative flow cytometric dot plots and the statistical analysis of the frequency of CX3CR1^+^Ly6C^−^ monocytes in AhR^△CD11c^ BMDCs (*n* = 4). (Q) mRNA expression of *Cx3cr1* in the peripheral blood of WT mice treated with or without GUANKE (*n* = 6). (R) Representative flow cytometric dot plots and the statistical analysis of the frequency of CX3CR1^+^Ly6C^−^ monocytes in the peripheral blood of WT mice treated with or without GUANKE (*n* = 6). (S) Representative flow cytometric dot plots and the statistical analysis of the frequency of CX3CR1^+^Ly6C^−^ monocytes in the peripheral blood of AhR^△CD11c^ mice treated with or without IAA (*n* = 5). Data are presented as mean ± SD. Statistical significance was determined by one‐way ANOVA with Tukey's post hoc test, except for (D‐I, K, and S), which were analyzed using two‐way ANOVA with Tukey's post hoc test. ^*^ *p* < 0.05, ^**^ *p* < 0.01, ^***^ *p* < 0.001. n.s., not significant.

Given that IAA is a well‐established ligand for the aryl hydrocarbon receptor (AhR) with known anti‐asthmatic properties, we initially hypothesized that GUANKE exerts its protective effects via the IAA‐AhR axis. However, GUANKE treatment did not increase *Ahr* expression in lung tissue (Figure 7A), and in vitro IAA stimulation markedly reduced *Ahr* mRNA levels in BMDCs (Figure 7B). To determine whether the protective effect of IAA requires AhR signaling in CD11c^+^ cells, we employed CD11c‐specific AhR knockout mice (*CD11c‐Cre* ^+^ *AhR* ^fl^/^fl^, hereinafter referred to as AhR^△CD11c^). Notably, IAA retained its capacity to inhibit OVA‐induced CD11b^+^ DC accumulation in AhR‐deficient BMDCs (Figure 7C). In agreement with these in vitro findings, in vivo IAA administration significantly ameliorated the asthmatic phenotype in AhR^△CD11c^ mice, as evidenced by restored cytokine homeostasis (Figure 7D–I) and marked suppression of CD11b^+^ DC accumulation and activation in the lungs (Figure 7J,K).

To explore the alternative mechanisms underlying this effect, which was preserved despite the absence of AhR signaling in CD11c^+^ cells, we performed RNA sequencing on OVA‐pulsed BMDCs. Transcriptomic profiling confirmed that IAA suppressed the OVA‐induced upregulation of *Ahr* and significantly downregulated critical DC development genes, including *Flt3*, *H2‐Aa*, *H2‐Ab1*, and *Csf1r* (Figure 7L). These transcriptomic findings indicated that IAA might be associated with broad changes in gene programs related to DC development and function, prompting us to further examine candidate pathways that might underlie the preserved effect of IAA in the absence of AhR signaling in CD11c^+^ cells. KEGG enrichment analysis highlighted the cytokine‐cytokine receptor interaction pathway, in which *Cx3cr1* was identified as a notably downregulated gene (Figure 7L,M). Given that lung CD11b^+^ DCs are partially replenished by circulating CX3CR1^+^Ly6C^−^ monocytic precursors [11], we hypothesized that IAA may affect DC accumulation by limiting this specific precursor pool. RT‐qPCR confirmed a significant downregulation of *Cx3cr1* mRNA expression in both WT and AhR^△CD11c^ BMDCs following IAA treatment (Figure 7N,O), which corresponded with an inhibited differentiation of CX3CR1^+^Ly6C^−^ monocytes in vitro (Figure 7P). Consistent with these findings, the abundance of CX3CR1^+^Ly6C^−^ monocytes was significantly reduced in the peripheral blood of GUANKE‐treated WT mice compared to the AAI group (Figure 7Q,R). Moreover, IAA administration significantly decreased the proportion of peripheral CX3CR1^+^Ly6C^−^ monocytes in both WT and AhR^△CD11c^ AAI mice (Figure 7S). Together, these results suggest that the reduction in pulmonary CD11b^+^ DC accumulation following IAA treatment is associated with decreased availability of circulating CX3CR1^+^Ly6C^−^ monocytic precursors, and that both effects are preserved after CD11c‐specific AhR deletion.

### 3.8 STAT3‐Jag1‐Notch4 Signaling Contributes to IAA‐Mediated Suppression of CX3CR1^+^Ly6C^−^ Precursor Generation

**FIGURE 8:**
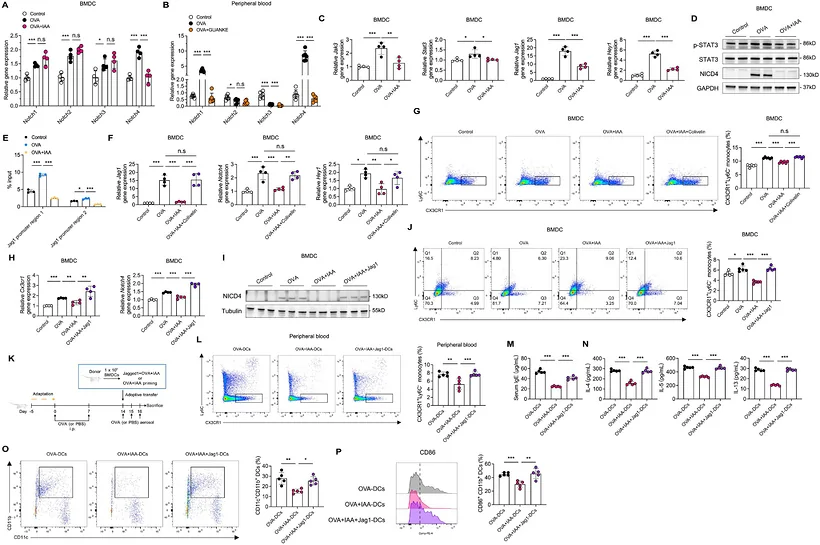
STAT3 activation and Jagged1‐mediated Notch reactivation partially reversed the effects of IAA on CX3CR1^+^Ly6C^−^ precursor differentiation. (A) mRNA expression of *Notch1*‐*Notch4* in control, OVA‐pulsed, and OVA+IAA‐treated BMDCs (*n* = 4). (B) mRNA expression of *Notch1*‐*Notch4* in peripheral blood from the control, OVA, and OVA+GUANKE groups (*n* = 6). (C) mRNA expression of *Jak3*, *Stat3*, *Jag1* and *Hey1* in BMDCs (*n* = 4). (D) Western blot analysis of p‐STAT3, STAT3, and NICD4 in BMDCs. (E) ChIP‐qPCR analysis of STAT3 occupancy at two distinct regions within the *Jag1* promoter in control, OVA‐pulsed, and OVA+IAA‐treated BMDCs. Enrichment is expressed as percentage of input (*n* = 3). (F‐G) BMDCs were treated with the STAT3 agonist colivelin to evaluate functional rescue of the pathway. mRNA expression of *Jag1*, *Notch4*, and *Hey1* is shown in (F). Representative flow cytometric plots and quantification of CX3CR1^+^Ly6C^−^ monocytes in BMDC cultures are shown in (G) (*n* = 4). (H‐J) BMDCs were treated with recombinant Jagged1 to reactivate downstream Notch signaling. mRNA expression of *Cx3cr1* and *Notch4* is shown in (H) (*n* = 4), representative western blot analysis of NICD4 is shown in (I), and representative flow cytometric plots and quantification of CX3CR1^+^Ly6C^−^ monocytes are shown in (J) (*n* = 5). (K) Experimental scheme of adoptive transfer. BMDCs pulsed with OVA and IAA in the presence or absence of recombinant Jagged1 and enriched by magnetic cell sorting. CD11c^+^ BMDCs (1×10^6^) were adoptively transferred into recipient mice 4 h before OVA challenge on day 14, followed by OVA aerosol challenge from days 14–16. Mice were sacrificed for analysis on day 17. (L) Representative flow cytometric dot plots and the statistical analysis of the frequency of CX3CR1^+^Ly6C^−^ monocytes in the peripheral blood from recipient mice (*n* = 5). (M) Serum IgE levels in recipient mice (*n* = 5). (N) IL‐4, IL‐5 and IL‐13 in lung homogenates of recipient mice (*n* = 5). (O) Representative flow cytometric dot plots and the statistical analysis of the frequency of CD11b^+^ DCs in lung tissue of recipient mice (*n* = 5). (P) Representative flow cytometry histogram (left) and percentages of CD86^+^ cells among CD11b^+^ DCs (right) detected in (O) (*n* = 5). Data are presented as mean ± SD. Statistical significance was determined by one‐way ANOVA followed by Tukey's post hoc test for all quantitative panels except panel E, which was analyzed using two‐way ANOVA followed by Tukey's post hoc test. ^*^ *p* < 0.05, ^**^ *p* < 0.01, ^***^ *p* < 0.001. n.s., not significant.

To identify signaling pathways associated with IAA‐mediated suppression of *Cx3cr1*, we further analyzed the transcriptomic profiles of OVA‐pulsed BMDCs treated with IAA. In addition to the reduction in *Cx3cr1*, RNA‐seq revealed coordinated decreases in components of the JAK‐STAT pathway (*Jak3*, *Stat3*) and the Notch pathway (*Jag1*, *Notch4*, *Hey1*) (Figure 7L,M). Among the Notch receptors, *Notch4* showed the most consistent reduction in IAA‐treated BMDCs and in the peripheral blood of GUANKE‐treated AAI mice (Figure 8A,B). RT‐qPCR further confirmed that IAA reduced the mRNA expression of *Jak3* and *Stat3*, together with parallel decreases in *Jag1*, *Notch4*, and *Hey1* (Figure 8C). Western blot analysis showed that IAA decreased p‐STAT3 abundance without altering total STAT3 levels and was accompanied by a reduction in NICD4 (Figure 8D). ChIP‐qPCR further demonstrated that OVA stimulation increased STAT3 occupancy at two predicted STAT3‐binding regions within the *Jag1* promoter, whereas IAA significantly reduced this OVA‐induced enrichment (Figure 8E). These findings provide promoter‐occupancy evidence linking suppression of STAT3 signaling to attenuation of the Jag1‐Notch4‐Hey1 axis.

To determine whether JAK3‐STAT3 signaling functionally contributes to this process, we performed rescue experiments using the STAT3 agonist colivelin. Compared with the OVA+IAA group, colivelin partially restored *Jag1*, *Notch4*, and *Hey1* expression (Figure 8F) and increased the proportion of CX3CR1^+^Ly6C^−^ monocytic precursors suppressed by IAA (Figure 8G). These rescue experiments support a functional contribution of STAT3 signaling to IAA‐mediated regulation of the Jag1‐Notch4 pathway and monocytic precursor differentiation.

We next assessed whether Jag1‐Notch4 signaling acts as a downstream effector of this pathway. Given the established role of Notch signaling in DC precursor differentiation [34], we reactivated this pathway by treating BMDCs with recombinant Jagged1 [35]. Jagged1 treatment counteracted the IAA‐induced reduction in *Cx3cr1* and *Notch4* mRNA expression and restored NICD4 protein abundance (Figure 8H,I). Flow cytometric analysis further showed that Jagged1‐mediated reactivation significantly increased the proportion of CX3CR1^+^Ly6C^−^ monocytes in vitro (Figure 8J), indicating that attenuation of Jag1‐Notch4 signaling contributes to the inhibitory effect of IAA on monocyte precursor differentiation.

To further evaluate the in vivo relevance of this pathway in allergic asthma, BMDCs were pulsed with OVA and treated with IAA in the presence or absence of recombinant Jagged1 before adoptive transfer to OVA‐challenged recipient mice (Figure 8K). Mice receiving OVA+IAA+Jag1‐DCs displayed restored frequencies of peripheral CX3CR1^+^Ly6C^−^ monocytes (Figure 8L), accompanied by increased serum IgE and elevated pulmonary IL‐4, IL‐5, and IL‐13 levels (Figure 8M,N). Jagged1 treatment also re‐established the recruitment and activation of pulmonary CD11b^+^ DCs (Figure 8O,P). Collectively, these findings support a functional contribution of JAK3‐STAT3 and Jag1‐Notch4 signaling to the effects of IAA on CX3CR1^+^Ly6C^−^ precursor generation and pulmonary CD11b^+^ DC replenishment during AAI.

## 4 Discussion

Modulation of the gut microbiota and its metabolites represents a promising therapeutic strategy for allergic asthma. Although microbiota‐derived indole derivatives have been implicated in the regulation of allergic immune responses [15, 18], their cell‐specific targets and mechanisms remain incompletely defined, particularly in asthma. In this study, we identified reduced circulating IAA as a metabolic alteration shared by AAI mice and patients with asthma. In the prophylactic AAI model, GUANKE supplementation increased intestinal IAA levels and restored circulating IAA in AAI mice, which was accompanied by marked reductions in airway mucus hypersecretion, pulmonary eosinophil infiltration, and Th2‐associated cytokine production. Importantly, therapeutic administration produced a similar protective profile and recovered circulating IAA after OVA challenge. Together, these findings identify microbiota‐associated IAA restoration as a consistent metabolic correlate of GUANKE‐mediated protection.

Although IAA can be synthesized from GUANKE in vitro, the contrasting results of the Abx and FMT experiments suggest that it may not be the principal source of restored IAA in vivo. FMT from GUANKE‐treated donors increased serum IAA and transferred protection against AAI, whereas direct GUANKE administration after broad‐spectrum Abx pretreatment failed to reproduce either effect. These findings support an association between GUANKE‐mediated protection and its interaction with the Abx‐sensitive resident microbial community, rather than autonomous IAA production. This interpretation is consistent with the evidence that exogenous probiotics can transiently reshape the metabolic activity of the resident microbiota without becoming dominant or persistent metabolic contributors [36, 37]. GUANKE treatment was also accompanied by enrichment of several *Bacteroides* taxa. Members of this lineage have been implicated in tryptophan metabolism and IAA‐related metabolic activity [38, 39, 40], and our recent work further linked *B. uniformis* to enhanced IAA production and protection against AAI [41]. Collectively, these observations support an association between GUANKE‐related changes in the resident microbiota and endogenous IAA restoration, although the specific taxa and metabolic pathways involved remain to be established.

CD11b^+^ DCs play a critical role in initiating Th2 responses and type 2 cytokine production in AAI [42, 43], and can amplify local inflammation through proinflammatory chemokines [8]. GUANKE reduced both the recruitment and activation of pulmonary CD11b^+^ DCs, providing a cellular correlate for the accompanying attenuation of Th2 inflammation. Although IAA has been shown to modulate DC subset differentiation and maturation in colitis [44], our findings extend this activity to allergic asthma. IAA directly limited CD11b^+^ DC accumulation, maturation, and Th2‐polarizing capacity in vitro, while adoptive transfer of IAA‐conditioned DCs attenuated Th2 inflammation in recipient mice. Direct IAA supplementation nevertheless produced broader immune changes than GUANKE, including recovery of IFN‐γ production, partial restoration of Foxp3^+^ Tregs, and suppression of IL‐17A in vivo, consistent with prior reports [41, 45]. The increase in Th17‐associated cytokines in the simplified DC‐T cell co‐culture system contrasted with the reduction in IL‐17A observed in vivo and may reflect the absence of systemic Treg‐mediated regulation. Thus, DC modulation may contribute importantly to the anti‐Th2 effect of IAA, while its overall activity likely involves broader immune networks in the intact host.

Extensive prior research has established IAA as an AhR ligand, and many of its reported protective effects have been attributed to AhR‐dependent regulation of epithelial barrier repair and Foxp3^+^ Treg differentiation [45, 46]. Here, however, IAA continued to suppress CX3CR1^+^Ly6C^−^ precursor generation in AhR‐deficient BMDCs and CD11c‐specific AhR knockout mice. This finding does not exclude potential AhR‐dependent effects of IAA in other pulmonary or immune cell populations, but suggests that IAA may engage distinct signaling programs depending on the cellular context. Transcriptomic and validation analyses instead revealed coordinated reductions in *Jak3*, *Stat3*, *Jag1*, *Notch4*, and *Hey1* expression, accompanied by lower p‐STAT3 and NICD4 protein abundance. In line with previous reports that STAT3 transcriptionally regulates *Jag1* [47, 48, 49], ChIP‐qPCR showed increased STAT3 occupancy at two regions of the *Jag1* promoter after OVA stimulation and reduced occupancy following IAA treatment. Pharmacological STAT3 activation partially restored Jag1‐Notch4‐Hey1 expression and CX3CR1^+^Ly6C^−^ precursor generation, supporting a functional contribution of STAT3 signaling to these IAA‐associated changes. Reports that structurally related indole compounds modulate JAK‐STAT signaling further support the biological plausibility of this mechanism [50, 51, 52].

Within this framework, Jag1‐Notch4‐Hey1 signaling is more likely to function as a downstream developmental checkpoint of STAT3 in monocyte precursor differentiation. Notch signaling is classically associated with T‐cell lineage commitment and lymphoid development [53], but it is also required for mucosal DC differentiation [34], and pulmonary CD11b^+^ DCs are replenished in part by circulating Ly6C^low^ monocytes [11, 54]. In this context, reduced *Cx3cr1* expression following Jag1‐Notch4 attenuation may limit the generation and availability of CX3CR1^+^Ly6C^−^ precursors. The ability of Jagged1 reactivation to restore precursor differentiation in vitro and to re‐establish pulmonary CD11b^+^ DC recruitment and Th2 inflammation after adoptive transfer provides functional support for this proposed regulatory relationship. Thus, these findings suggest that the effects of IAA extend beyond the modulation of mature DC function to earlier stages of monocyte‐to‐DC differentiation, with STAT3‐Jag1‐Notch4 signaling potentially linking reduced CX3CR1^+^Ly6C^−^ precursor generation to diminished pulmonary CD11b^+^ DC replenishment.

The lower circulating IAA levels detected in patients with asthma provide clinical support for the association between altered IAA‐related metabolism and asthma, although the cross‐sectional design does not establish causality or directly demonstrate that GUANKE restores IAA in humans. This finding is consistent with alterations in microbial tryptophan‐related metabolites in wheezing preschool children [55], indole derivatives within an allergy‐associated molecular network [17], and the enrichment of indole‐related metabolites in healthy or low‐asthma indoor environments [56, 57]. These observations collectively suggest a link between microbiota‐associated indole metabolism and allergic phenotypes. However, the immunomodulatory properties of IAA exhibit a distinct functional dichotomy when evaluated across divergent pathological conditions. Elevated circulating IAA has been associated with mortality in severe community‐acquired pneumonia [58], while its accumulation as a uremic toxin has been linked to cardiovascular dysfunction in chronic kidney disease [59]. Therefore, clinical translation should focus on controlled restoration of physiological IAA‐related metabolism and appropriate patient stratification, rather than nonspecific systemic IAA supplementation.

Several limitations exist in the current study. First, mechanistic evidence was obtained primarily from an OVA‐induced murine model, while the clinical analysis was cross‐sectional and involved a relatively small cohort. Larger cohorts and validation in relevant human immune cells are therefore needed. Second, although exogenous IAA reproduced key features of GUANKE‐mediated protection, its necessity for GUANKE efficacy remains to be established through IAA‐specific loss‐of‐function studies. Third, the Abx, FMT, and full‐length 16S rRNA sequencing data support a microbiota‐dependent contribution to IAA restoration but do not identify the responsible strains, bacterial genes, or biosynthetic pathways. Finally, pharmacological rescue and ChIP‐qPCR implicate STAT3‐Jag1‐Notch4 signaling, but the genetic requirement for STAT3, the functional relevance of the predicted STAT3‐binding regions in the *Jag1* promoter, and the direct molecular target of IAA remain unresolved.

In conclusion, GUANKE alleviates AAI in association with microbiota‐dependent restoration of circulating IAA. Our findings connect IAA to the regulation of CX3CR1^+^Ly6C^−^ monocytic precursors and pulmonary CD11b^+^ DC responses and support a contributing role for STAT3‐Jag1‐Notch4 signaling, while indicating that these cellular effects are preserved after CD11c‐specific AhR deletion. Together with the lower circulating IAA observed in patients with asthma, these results provide a basis for further mechanistic and translational evaluation of microbiota‐related IAA metabolism in allergic asthma.

## Author Contributions

YJH: Writing – original draft, Methodology, Investigation, Conceptualization. JM: Validation, Investigation, Formal analysis. ZZ: Resources, Investigation, Validation, Data Curation. XZ: Investigation. ZY: Investigation. KY: Investigation. YMH: Data curation. LS: Investigation. XW: Resources, Data Curation, Supervision, Funding acquisition. JX: Resources, Funding acquisition. ZR: Writing – review & editing, Resources, Funding acquisition, Conceptualization.

## Funding

This work was supported by grants from Detection Technology Reserve for Highly Pathogenic Pathogens Posing Biosafety Threats (Grant Nos. 2026NITFID512, 01058652100409004) and the National Institute for Communicable Disease Control and Prevention, Chinese Center for Disease Control and Prevention (Grant No. 33054 and 29172), the National Natural Science Foundation of China (Grant No. 82202005).

## Conflicts of Interest

JX declares a financial conflict of interest arising from the transfer of the GUANKE strain patent to Maiyata Biopharmaceuticals Inc. The remaining authors disclose no potential conflicts.

## Supporting information




**Supporting File**: advs77783‐sup‐0001‐SuppMat.docx.

## Data Availability

The 16S rRNA gene sequencing raw sequence reads (fastq) and RNA‐seq sequencing data produced in this study are available in the NCBI Sequence Read Archive under accession projects PRJNA1333678 and PRJNA1334023. The targeted serum tryptophan metabolomics data from the human cohort have been deposited in the OMIX repository of the National Genomics Data Center, China National Center for Bioinformation/Beijing Institute of Genomics, Chinese Academy of Sciences (https://ngdc.cncb.ac.cn/omix; accession number OMIX018914).
