# Supplementary material for: Lactiplantibacillus Plantarum GUANKE Alleviates Allergic Asthma by Restricting Monocyte‐to‐DC Differentiation Via Microbiota‐Associated IAA
Source: Adv Sci (Weinh). 2026 Sep 16:e23466. Online ahead of print. doi: 10.1002/advs.202523466 (PMC13583224; doi:10.1002/advs.202523466)
Supplement: Supplementary file 1 [file ADVS-9999-e23466-s001.docx]

**Table S1. Demographic and clinical characteristics of patients with asthma and healthy controls related to Figure 3**

| **Parameters** | **Healthy controls (*n* = 28)** | **Patients with Asthma (*n* = 28)** |
| --- | --- | --- |
| Age, years | 50.0 (29.5-55.0) | 43.0 (33.5-54.5) |
| Gender (male/female) | 8/20 | 12/16 |
| FEV_1_ (L) | NA | 3.16 (2.77-3.77)^a^ |
| Total IgE (IU/mL) | NA | 87.1 (20.0-197.0)^b^ |
| Blood eosinophils (×10^9^/L) | 0.08 (0.05-0.18) | 0.12 (0.09-0.20) |

Data are presented as median (Q1–Q3) for continuous variables and as n for categorical variables. ᵃ FEV_1_ data were available for 15 patients with asthma. ᵇ Serum total IgE data were available for 27 patients with asthma. FEV_1_, forced expiratory volume in 1 s; IgE, immunoglobulin E; NA, not available.

**Table S2. Primers used for quantitative real-time PCR analysis**

| Species/genes | Primer sequence |
| --- | --- |
| m*-Ccl2* Forward | 5’-GATGCAGTTAACGCCCCACT-3’ |
| m-*Ccl2* Reverse | 5’-ACCCATTCCTTCTTGGGGTC-3’ |
| m-*Ccl8* Forward | 5’-GCCAGATAAGGCTCCAGTCA-3’ |
| m-*Ccl8* Reverse | 5’-TGCCTGGAGAAGATTAGGGGA-3’ |
| m-*Ccl20* Forward | 5’-CAGGTCTCCTCATGGCTTTGC-3’ |
| m-*Ccl20* Reverse | 5’-AATCCTTCCACTAAGCGCCC-3’ |
| m-*Ccl17* Forward | 5’-CAATGTAGGCCGAGAGTGCT-3’ |
| m-*Ccl17* Reverse | 5’-CCTGGAACACTCCACTGAGG-3’ |
| m-*Ccl22* Forward | 5’-TTCTCTGCCCCACACCTTTG-3’ |
| m-*Ccl22* Reverse | 5’-GCAGCTCCCACAACCATCTA-3’ |
| m-*Cx3cr1* Forward | 5’-GAGTATGACGATTCTGCTGAGG-3’ |
| m-*Cx3cr1* Reverse | 5’-CAGACCGAACGTGAAGACGAG-3’ |
| m-*Notch1* Forward | 5’-GCGAAGTGGACATTGACGAG-3’ |
| m-*Notch1* Reverse | 5’-GCTGGCACAGGCAGGTAAAG-3’ |
| m-*Notch2* Forward | 5’-AGCAGGAGCAGGAGGTGATA-3’ |
| m-*Notch2* Reverse | 5’-TGGGCGTTTCTTGGACTCTC-3’ |
| m-*Notch3* Forward | 5’-ACTCCTCCTCAGGGAGATGC-3’ |
| m-*Notch3* Reverse | 5’-GTGGGGTGAAGCCATCAGG-3’ |
| m-*Notch4* Forward | 5’-AGGCCATGTTCTCCAGTCAC-3’ |
| m-*Notch4* Reverse | 5’-TTTCCATCTCACCCTTTAGTCCC-3’ |
| m-*Jag1* Forward | 5’-CCTCGGGTCAGTTTGAGCTG-3’ |
| m-*Jag1* Reverse | 5’-CCTTGAGGCACACTTTGAAGTA-3’ |
| m-*Hey1* Forward | 5’-GCGCGGACGAGAATGGAAA-3’ |
| m-*Hey1* Reverse | 5’-TCAGGTGATCCACAGTCATCTG-3’ |
| m-*Jak3* Forward | 5’-AGGTCGTGGATGGTGAGACACA-3’ |
| m-*Jak3* Reverse | 5’-AACACCAGGTGCGGGTAGGATA-3’ |
| m-*Stat3* Forward | 5’-CAATACCATTGACCTGCCGAT-3’ |

**Table S2 (continued)**

| Species/genes | Primer sequence |
| --- | --- |
| m-*Stat3* Reverse | 5’-GAGCGACTCAAACTGCCCT-3’ |
| m-*Ahr* Forward | 5’-AGCCGGTGCAGAAAACAGTAA-3’ |
| m-*Ahr* Reverse | 5’-AGGCGGTCTAACTCTGTGTTC-3’ |
| m-*actin* Forward | 5’-ATATCGCTGCGCTGGTCG-3’ |
| m-*actin* Reverse | 5’-TTCCCACCATCACACCCTGG-3’ |

m: mouse

**Table S3. Primers used for ChIP-qPCR analysis**

| Species/genes | Primer sequence |
| --- | --- |
| m-*Jag1* promoter region 1 Forward | 5’-CTCAACACGCGGAACAAGGC-3’ |
| m-*Jag1* promoter region 1 Reverse | 5’-CCAACAACCCGGAGGAAAGG-3’ |
| m-*Jag1* promoter region 2 Forward | 5’-CTTCCGCCTTCGGAGCCG-3’ |
| m-*Jag1* promoter region 2 Reverse | 5’-CAGCACAGGGCTCTGGGC-3’ |

m: mouse

**Figure S1**

**Figure S1. Gating strategy.**

(A-B) Gating strategy and representative flow cytometric plot for the analysis of CD11b^+^ DCs in the lungs (A) of AAI mice or in BMDCs (B).
(C) Gating strategy and representative flow cytometric plot for the analysis of CD4^+^CD25^+^Foxp3^+^ Tregs in the spleens of AAI mice.
(D-E) Gating strategy and representative flow cytometric plot for the analysis of CX3CR1^+^Ly6C^-^ monocytes in the peripheral blood (D) of AAI mice or in BMDCs (E).

(F) Gating strategy and representative flow cytometric plot for the analysis of CD4^+^IL-4^+^ and CD4^+^IFN-γ^+^ T cells of CD4^+^ T cells co-cultured with different BMDCs.

**Figure S2**

**Figure S2. Top-ranked species-level taxa associated with GUANKE-induced gut microbiota remodeling.** Species-level differential analysis was performed across the Control, OVA, and OVA+GUANKE groups, and the 10 top-ranked species with the smallest nominal *P* values were selected for visualization. Relative abundance of each species in individual samples is shown for (A) *Marvinbryantia formatexigens*, (B) *Hominibacterium faecale*, (C) *Adlercreutzia murintestinalis*, (D) *Porphyromonadaceae bacterium UBA7139*, (E) *Bacteroides uniformis*, (F) *Duncaniella freteri*, (G) *Candidatus Formimonas warabiya*, (H) *Bacteroides faecichinchillae*, (I) *Bacteroides caecimuris*, and (J) *Bacteroides acidifaciens*. *n*=6 per group, and horizontal bars indicate the mean. *P* values shown in each panel represent the raw *P* values from the overall comparison among the three groups.

**Figure S3**

**Figure S3. GUANKE alleviated OVA-induced allergic asthma after 7 days of treatment.**

(A) Experimental schema for B-I. Mice were administered GUANKE (1×10^9^ CFU) or PBS via daily oral gavage for 7 consecutive days following the final OVA challenge. Mice received OVA sensitization on days 0 and 7 (with some groups not sensitized), followed by OVA challenge on days 14 to 16.

(B) Representative flow cytometric dot plots for showing the proportions of eosinophils, macrophages, neutrophils and monocytes in lung tissue.

(C) Percentages of inflammatory cells among lung CD45^+^ leukocytes quantified from panel (B).

(D) Serum IgE.

(E-G) IL-5, IL-17A and IFN-γ in lung homogenates.

(H) Representative flow cytometric dot plots and the statistical analysis of the frequency of CD11b^+^ DCs in lungs from mice.

(I) Serum IAA. Data are presented as mean ± SD. *n*=6 per group. Statistical significance was determined by one-way ANOVA with Tukey’s post hoc test. **P* < 0.05, ***P* < 0.01, ****P* < 0.001. n.s., not significant.

**Figure S4**

Figure S4. Cell viability monitored by LDH release assay. Data are presented as mean ± SD. Cell viability (%) = 100% - LDH relative release (%). Statistical significance was determined by unpaired Student’s *t-*test. n.s., not significant.
